# Systems biology approaches to understanding the cause and treatment of heart, lung, blood, and sleep disorders

**DOI:** 10.3389/fphys.2014.00107

**Published:** 2014-03-28

**Authors:** Raimond L. Winslow

**Affiliations:** Biomedical Engineering, School of Medicine, Institute for Computational Medicine, The Johns Hopkins UniversityBaltimore, MD, USA

**Keywords:** computational medicine, pulmonary models, cardiac models, immune system models, sepsis models, micro-RNA networks

Development of powerful new high-throughput technologies for probing the transcriptome, proteome, and metabolome is driving the rapid acquisition of information on the function of molecular systems. One of the greatest challenges that we now confront is to understand how behavior at the level of genome, proteome, and metabolome shapes physiological function at the level of cell, tissue and organ in both health and disease. Because of the inherent complexity of biological systems, the development, analysis, and validation of integrative computational models based directly on experimental data is necessary to achieve this understanding. This approach, known as *systems biology*, integrates computational and experimental approaches through iterative development of mathematical models and experimental validation and testing. The combination of these approaches allows for a mechanistic understanding of the function of complex biological systems in health, and their dysfunction in disease.

In 2006, the National Heart, Lung, and Blood Institute (NHLBI) announced the Exploratory Program in Systems Biology, followed in 2010 by the NHLBI Systems Biology Collaborations. The goal of these programs is to support collaborative teams of investigators in using experimental and computational strategies to integrate the component parts of biological networks and pathways into computational models that are based firmly on and validated using experimental data. These validated models are then applied to gain insights into the mechanisms of altered system function in disease, to generate novel hypotheses regarding these mechanisms that can be tested experimentally, and to then use the results of experiments to refine the models. This Research Topic is comprised of articles by researchers funded by this NHLBI program. One set of publications focus on *lung function in health and disease*. Lauzon et al. (2012) present a multi-scale model of lung function spanning molecular (force production by actin-myosin proteins), cellular (regulation of force production by calcium signaling), tissue (smooth muscle contraction coupled with the biomechanics of airway narrowing), and organ scales (mechanical lung impedance). The model provides a new tool for investigating airway hyper responsiveness in the setting of asthma and other lung diseases. O'Connor et al. (2012) present a closed-loop model of neural control of breathing that includes a neural network model of the respiratory pattern generator that drives a systems-level model of lung contraction with feedback to the respiratory pattern generator driven by mechano- and baro-receptor reflexes. The model provides a framework for studying cough disorders. Segers et al. (2012) present results from an experimental study aimed at identifying the role of different neuronal populations in regulating fricative cough. Finally, Fallahi-Sichani et al. (2012) present a multi-scale model of the lung immune response to *Mycobacterium tuberculosis* and use the model to examine the ways in which the NF-kB signaling pathway regulates inflammatory responses. Another set of publications focus on *blood disease*. Dick et al. (2012) develop a multi-scale model of acute inflammatory disease, such as sepsis, that describes baro-, chemo-, and cytokine-reflex control of cardio-pulmonary function mediated by the nucleus *tractus solitarius* (nTS). They present the model-motivated hypothesis that the dis-regulation of cardio-respiratory patterns in the setting of sepsis results from expression of cytokines in the nTS. Goldman et al. (2012) develop models of the erythrocyte signaling pathways that control ATP release as a function of hemoglobin oxygen saturation. These models provide a new framework for studying regulation of microvascular perfusion distribution. Diamond et al. (2013) resent a modeling approach that combines fluid-dynamics simulation of blood flow in the presence of a developing thrombus, which drives formation of a boundary layer of soluble agonists that drives blood platelet motion and binding to the vessel wall. Platelet activation state is driven by the history of intracellular calcium concentration as determined using a neural network model. This work stands out in terms of the different biological scales that are modeled, the different computational approaches employed at each of these scales, and the ability to personalize the model and predict patient-specific responses. Ghonaim et al. (2013) present results from a combined experimental and modeling study of the relationship between hemoglobin oxygen saturation, erythrocyte capillary transit times, and stimulated ATP release. They use a novel experimental approach that uses micro-patterned plastic/glass substrates and a flow chamber to measure tissue hemoglobin oxygen saturation level in response to different patterns of tissue oxygen exposure. Experimental and modeling results demonstrate that erythrocyte ATP release is best stimulated by exposing a larger number of capillaries and endothelial cells to the oxygen signal. The paper by Lemons et al. (2013) is focused on the *regulation of protein-protein interaction networks*. They propose, given the large number of protein-protein interactions regulated by micro-RNAs (miRNAs), that miRNA screening may provide key insights into the specific gene-protein networks that are dis-regulated in disease. Using miRNA screening and analytical approaches for identifying miRNA targets, these investigators identified two miRNAs that are up-regulated in human heart failure, and that inhibit SERCA2. This novel finding is supported by the fact that down-regulation of SERCA2 is a well-known hall-mark of heart failure. Finally, Gauthier et al. (2012) develop a novel, highly *integrative model of the guinea pig ventricular myocyte* describing the key properties of calcium-induced calcium-release that drives cardiac muscle contraction. Using the model, they show that even subtle changes of action potential shape (as is common in many different types of heart disease) can lead to significant changes in the timing and amplitude of intracellular calcium transients and force generation in the heart.

This body of work illustrates the exciting, new, emerging directions that research on heart, lung, blood, and sleep disorders is taking. This work shares common features. Each takes a systems biology approach in which experimental data are used to formulate and refine computational models. Each of the models presented are multi-scale in that they describe function across different levels of biological organization—ranging from molecular interactions to the function of cells, tissue, and in some cases organs. Some models couple different computational methods between scales. In every case, this dove-tailing of experiment and modeling is leading to novel, deep insights into the nature of heart, lung, and blood disease.
